# Using linked administrative data to evaluate and improve the quality of end-of-life care in nursing homes.

**DOI:** 10.23889/ijpds.v7i3.2006

**Published:** 2022-08-25

**Authors:** Colleen Webber, Christina Milani, Anna Clarke, Sarina R Isenberg, James Downar, Daniel Kobewka, Amy Hsu, Jenny Lau, Aynharan Sinnarajah, Jessica Simon, Kaitlyn Boese, Amit Arya, Breffni Hannon, Rhiannon Roberts, Luke Turcotte, Michelle Howard, Colleen Maxwell, Peter Tanuseputro

**Affiliations:** 1 Ottawa Hospital Research Institute; 2 ICES; 3 Bruyere Research Institute; 4 University of Ottawa; 5 University of Toronto; 6 Lakeridge Health; 7 Alberta Health Services; 8 The Ottawa Hospital; 9 North York General Hospital; 10 University Health Network; 11 University of Waterloo; 12 McMaster University

## Objectives

Prescribing of symptom management medications may reflect the quality of end-of-life care provided to nursing home residents who are nearing death. The objective of this study was to examine variations in the prescribing of end-of-life symptom management medications in nursing home residents in the last 14 days of life.

## Approach

TREs provide secure facilities to analyse confidential personal data, with staff checking outputs for disclosure risk before publication. Artificial intelligence (AI) has high potential to improve the linking and analysis of population data, and TREs are well suited to supporting AI modelling. However, TRE governance focuses on classical statistical data analysis. The size and complexity of AI models presents significant challenges for the disclosure-checking process. Models may be susceptible to external hacking: complicated methods to reverse engineer the learning process to find out about the data used for training, with more potential to lead to re-identification than conventional statistical methods.

## Results

There were 55,029 deaths across 626 nursing homes. Overall, 64.8% of residents received at least one end-of-life symptom management medication. The proportion of dying residents who received ≥1 end-of-life medication ranged from 37.6% in quintile 1, 59.8% in quintile 2, 69.1% in quintile 3, 74.8% in quintile 4, and 82.9% in quintile 5. Opioids were the most commonly prescribed medications, with an average of 62.2% of residents receiving a prescription (35.9% to 81.2% across the quintiles). Nursing home residents that resided in homes in the lowest prescribing quintile were older and more likely to be Allophones (first language not English or French). Low prescribing homes were also larger, with a higher number of beds, and were more likely to be in rural areas.

## Conclusion

The observed variations in the prescribing of medications to manage end-of-life symptoms in nursing home residents raises concerns that some residents may have received inadequate end-of-life symptom management. Prescription data may provide an opportunity to rapidly evaluate the quality of end-of-life care in nursing homes at a population level.

